# Supplementary material for: Empirical methods for controlling false positives and estimating confidence in ChIP-Seq peaks
Source: BMC Bioinformatics. 2008 Dec 5;9:523. doi: 10.1186/1471-2105-9-523 (PMC2628906; doi:10.1186/1471-2105-9-523)
Supplement: Additional File 1 — A variety of html documents from the USeq web site detailing the available applications, their best usage, output file type descriptions, command line menus, etc. [file 1471-2105-9-523-S1.zip › USeqUserGuides/JavaDoc.zip!/JavaDoc/util/gen/class-use/RankedFloatArray.html]

Uses of Class util.gen.RankedFloatArray


|  |  |  |
| --- | --- | --- |
| **Overview** \| **Package** \| **Class** \| **Use** \| **Tree** \| **Deprecated** \| **Index** \| **Help** | |  |
| PREV   NEXT | **FRAMES**    **NO FRAMES**     **All Classes** |  |


---


## **Uses of Class util.gen.RankedFloatArray**

| Packages that use RankedFloatArray | |
| --- | --- |
| **expr** | Classes and tools related to expression array analysis. |
| **util.gen** | Variety of helper methods for Email, IO, Encryption, HTML, Swing... |

Uses of RankedFloatArray in expr

| Methods in expr that return RankedFloatArray | |
| --- | --- |
| `RankedFloatArray` | `ExpressedGene.getRfa()` |

| Methods in expr with parameters of type RankedFloatArray | |
| --- | --- |
| `void` | `ExpressedGene.setRfa(RankedFloatArray rfa)` |

Uses of RankedFloatArray in util.gen

| Methods in util.gen that return RankedFloatArray | |
| --- | --- |
| `RankedFloatArray` | `SpearmanCorrelation.getARFA()` |
| `RankedFloatArray` | `SpearmanCorrelation.getBRFA()` |
| `RankedFloatArray` | `SpearmanCorrelation.rank(float[] f)` |
| `RankedFloatArray` | `SpearmanCorrelation.rankSamples(RankSample[] rs)`             Ranks a sorted array of RankSample based on value. |

| Methods in util.gen with parameters of type RankedFloatArray | |
| --- | --- |
| `void` | `SpearmanCorrelation.setARFA(RankedFloatArray arfa)` |
| `void` | `SpearmanCorrelation.setBRFA(RankedFloatArray brfa)` |
| `double` | `SpearmanCorrelation.spearmanCorrelationCoefficient(RankedFloatArray aRFA, RankedFloatArray bRFA)`             Returns a Spearman Rank Correlation Coefficient (rho) between the two arrays. |

---


|  |  |  |
| --- | --- | --- |
| **Overview** \| **Package** \| **Class** \| **Use** \| **Tree** \| **Deprecated** \| **Index** \| **Help** | |  |
| PREV   NEXT | **FRAMES**    **NO FRAMES**     **All Classes** |  |


---
